# YOGA MAY HAVE HEALTH BENEFITS FOR PEOPLE WITH CHRONIC NON-SPECIFIC LOWER BACK PAIN

**Published:** 2017-04

A new systematic review, published in the Cochrane Library today, suggests that yoga may lead to a reduction in pain and functional ability in people with chronic non-specific lower back pain over the short term, compared with no exercise. However, researchers advise that more studies are needed to provide information on long-term effects.

Lower back pain is a common health problem, and is usually treated with self-care and over-the-counter medication. For some people it may last for three months or more, and at this point it is considered “chronic”. Back pain is sometimes associated with a disease or condition, but the vast majority of lower back pain cases have an unknown cause, and as a result are described as non-specific. Current guidelines state that exercise therapy may be beneficial, and in particular yoga is sometimes used as a treatment.

Yoga has gained global popularity as a form of mind-body exercise, with general life-style benefits, and recent studies have investigated the potential of yoga to relieve the symptoms of lower back related problems.

A new Cochrane Review summarizes the results of 12 randomized trials from 1,080 men and women with an average age between 34 and 48 years old. The trials were conducted in India, the UK, and the US. All participants had chronic non-specific lower back pain.

The Cochrane researchers included studies that compared practising yoga in a class to not doing any back-focused exercise, or to other forms of exercise. Seven studies compared yoga with no exercise, three studies compared yoga with back-focused exercise, or added yoga for a back-focused exercise programme. Two studies compared yoga with two other forms of control group: no exercise or a self-care book. All yoga interventions used were specifically designed for treatment of lower back pain, and were provided by experienced and qualified teachers.

The Review found that compared to no exercise, practising yoga might improve back-related function and may also reduce symptoms of lower back pain by a small amount in the first six to twelve months, although the effect was consistently less than that judged to be clinically important. However, larger and more robust studies with longer follow up are needed to draw any firm conclusions about the long-term health effects of yoga.

However, yoga may cause an increase in back pain in some people. About 5% more yoga participants experienced increased back pain, although this may be similar to the risk of having side effects from other back-focused exercise.

Lead Cochrane author, Susan Wieland from Cochrane Complementary Medicine at the Center for Integrative Medicine, University of Maryland School of Medicine, Maryland, commented, “Our findings suggest that yoga exercise may lead to reducing the symptoms of lower back pain by a small amount, but the results have come from studies with a short follow up. At the moment we only have low to moderate quality evidence for the effects of yoga before six months as a type of exercise for helping people with chronic lower back pain. The yoga exercises practised in the studies were developed for low back pain and people should also remember that in each of the studies we reviewed, the yoga classes were led by experienced practitioners. The findings of this Cochrane Review will help people make more informed choices about their future treatment options.”

*Full citation: Wieland LS, Skoetz N, Pilkington K, Vempati R, D’Adamo CR, Berman BM. Yoga treatment for chronic non-specific low-back pain. Cochrane Database of Systematic Reviews 2013, Issue 7. Art. No.: CD010671. DOI: 10.1002/14651858. CD010671*.

*Copyright © 2017 The Cochrane Collaboration. Published by John Wiley & Sons, Ltd., reproduced with permission*.

